# Associations between communicator gender and animal avatar appearance: behavioral and eye-tracking evidence from animal avatar communication

**DOI:** 10.3389/fpsyg.2026.1936888

**Published:** 2026-10-08

**Authors:** Yu Han, Hayato Watanabe, Atsushi Shimojo, Koichi Yokosawa

**Affiliations:** 1 Graduate School of Health Sciences, Hokkaido University, Sapporo, Japan; 2 Faculty of Health Sciences, Hokkaido University, Sapporo, Japan; 3 Center for Information and Neural Networks, Advanced ICT Research Institute, National Institute of Information and Communications Technology, Suita, Japan

**Keywords:** avatar appearance, avatar-mediated communication, communicator gender, eye tracking, gaze allocation

## Abstract

Avatar-mediated communication is often used to reduce interpersonal bias by altering avatar appearance. However, information from the communicator may also affect how the avatar is perceived. We examined whether vocal characteristics related to communicator gender are associated with animal avatar appearance in an avatar identification task. Forty participants were assigned to four groups (*N* = 10 per group) defined by actor gender (female vs. male) and real-avatar type (cat vs. dog). Participants completed a dialogue task while viewing two avatars side by side. One avatar mirrored the actor’s facial and head movements in real time (real avatar), whereas the other showed pre-recorded, non-contingent movements (fake avatar). Gaze allocation was recorded throughout, and participants identified the real communication partner after the conversation. Identification outcomes (correct identification or misidentification) were analyzed using binary logistic regression with actor gender, real-avatar type, and their interaction. Fixation to the fake avatar was quantified across three time segments and analyzed using a mixed-design analysis of variance crossing actor gender, real-avatar type, and time segment. The actor gender × real-avatar type interaction was not significant (*p* = 0.075), although correct identification was 8/10 in each of the other three groups but only 2/10 in the female actor × dog avatar group. Fixation to the fake avatar was significantly greater in this group (*p* < 0.001). Participants in this group tended to identify the alternative cat avatar as the communication partner. These findings suggest that vocal characteristics and avatar appearance may interact to influence perception during avatar-mediated communication.

## Introduction

Avatar-mediated communication allows communicators to interact through visually altered representations, such as virtual faces animated by real-time facial tracking (Saragih et al., 2011). Because these representations can alter or obscure the communicator’s real-world visual identity cues, avatar-mediated communication may reduce reliance on appearance-based judgments.

In immersive virtual reality (VR), avatars differing from users in race have been associated with reductions in implicit racial bias (Peck et al., 2013), and comparable reductions in implicit age bias have been reported when users embody avatars of a different age (Banakou et al., 2018). Avatar use may also support selective identity disclosure: in an interview study, people with disabilities described using avatars strategically to represent, modify, or withhold disability-related information depending on the social context (Mack et al., 2023).

Although avatars can obscure a communicator’s real appearance, they do not eliminate visually based social judgments. Instead, the appearance of the avatar may itself provide cues from which impressions of the represented individual are formed. For example, variations in avatar appearance have been shown to influence perceptions of credibility, similarity, and interpersonal attraction (Nowak and Rauh, 2005). Substantial agreement has been reported in judgments of avatar trustworthiness, and people are more willing to trust individuals represented by avatars that appear trustworthy, even though these impressions are unrelated to the actual trustworthiness of the avatar creators (Machneva et al., 2022). Observers can also infer aspects of users’ personalities from their avatars, suggesting that avatar features may be interpreted as information about the person they represent (Fong and Mar, 2015). In addition, participants in an avatar-based interaction study maintained greater interpersonal distance from avatars with an out-group ethnic appearance than from in-group avatars (Menshikova et al., 2018). Avatar appearance has also been shown to influence communication itself, with differences in the degree of avatar deformation affecting how others communicated with avatar users across both formal and informal situations (Kobayashi et al., 2021). Together, these findings suggest that replacing a communicator’s real appearance with an avatar may remove some physical identity cues while introducing new visual cues that influence how the communicator is perceived.

Non-human avatars may obscure more social information about the communicator, while their distinctive appearance can still influence how information is perceived during communication. Zhang et al. (2024) found that relatively cute animal avatars facilitated emotion perception. At the same time, the species-specific appearance of animal avatars may even provide new cues from which characteristics of the person behind the avatar are inferred. Murakami et al. (2024) compared neural responses to cat and dog avatars before and after participants communicated with a female actor through either avatar. The results revealed that neural responses changed after communication with the cat avatar, whereas no comparable change was observed for the dog avatar (Murakami et al., 2024). Because the communicator was female in both avatar conditions, it remained unclear whether this cat-related response change reflected a general difference between cat and dog avatars or whether it was related to the pairing of a female communicator with a cat avatar. The latter possibility is consistent with cultural associations between cats and femininity and with gendered perceptions regarding cats and cat ownership (Dupuis and Girme, 2024; Lewis, 2024; Tu et al., 2024). Thus, the cat-related neural response change may have reflected an association between the female communicator and the cat avatar rather than a general difference between cat and dog avatars.

Building on the finding reported by Murakami et al. (2024), the present study examined whether gender-related information conveyed by the communicator’s voice may be associated with animal-avatar appearance during avatar-mediated communication, with particular focus on the possible association between a female communicator and a cat avatar. During an avatar-mediated dialogue, participants viewed cat and dog avatars simultaneously, one controlled in real time by the actor (real avatar) and the other displaying prerecorded, non-contingent movements (fake avatar). Participants then identified which avatar had been controlled by the communication partner. Actor Gender (male/female) and the Real-Avatar type (cat/dog) were varied across groups. In accord with the possibility that cat avatars may be more readily associated with female communicators, we expected more frequent misidentification of the fake cat avatar and greater fixation to it when the female actor controlled the dog avatar. In contrast, less frequent misidentification was expected when the female actor controlled the cat avatar or when the actor was male. This prediction was expected to depend on communicator gender. If the observed bias simply reflected general perceptual or stimulus-related differences between the cat and dog avatars, similar effects would be expected across male- and female-communicator conditions. In contrast, a gender-related association would be expected to produce a more selective pattern involving the female communicator.

Eye tracking may provide a sensitive measure of social expectations and biases that are not fully reflected in overt responses. Previous studies have shown that social biases can shape visual attention to faces, with eye-movement patterns reflecting differences in implicit and explicit prejudicial attitudes that may not be fully captured by overt responses (Hansen et al., 2015). Guerra et al. (2021) further demonstrated that gender-stereotypical information elicited anticipatory eye movements, although these gaze effects were not associated with explicit gender attitudes. In an avatar-mediated AI interview setting, Lau et al. (2026) found greater attention to an avatar’s face under racial mismatch, whereas identity matching did not significantly affect self-reported trust. Together, these findings suggest that gaze allocation can reveal social-cognitive effects that may not be clearly captured by explicit judgments. Eye tracking was therefore used in the present study to examine whether partner identification was accompanied by corresponding differences in gaze allocation during the dialogue.

## Method

### Participants

A total of 40 adults participated in the study (15 men, 25 women; mean age ± standard deviation = 26.4 ± 6.4 years). All participants reported normal or corrected-to-normal vision, and no history of neurological or psychiatric disorders. Participants were assigned to one of four groups (*N* = 10 per group) in a 2 × 2 design crossing Actor Gender (male, female) and Real-Avatar type (cat, dog) (Table 1). The total sample size was evaluated using G*Power 3.1.9.7 (Faul et al., 2009) for a mixed-design analysis of variance (ANOVA) with a 2 × 2 between-subjects design and a three-level within-subject factor (α = 0.05, power = 0.80), assuming relatively large effects (ηp^2^ > 0.20), and considering evidence that variance in area-of-interest (AOI)-based measures decreases efficiently up to n ≈ 40 (Hoogerbrugge et al., 2025). A one-way ANOVA showed no significant differences in age among the four groups, *F* (3, 36) = 1.37, *p* = 0.267.

**Table 1:** Demographics of four experimental groups (Actor Gender × Real-Avatar type).

Group (Actor Gender × Real-Avatar type)	N	Men: women	Age (years, M ± SD)
Male actor × cat	10	4:6	28.4 ± 6.8
Male actor × dog	10	4:6	27.8 ± 7.5
Female actor × cat	10	4:6	26.2 ± 7.0
Female actor × dog	10	3:7	23.2 ± 2.6

Demographic characteristics by group are presented in Table 1. The study was approved by the Ethics Committee of the Faculty of Health Sciences, Hokkaido University (Approval No. 23–41-1; research project title: Eye Movement Investigation During Communication), and written informed consent was obtained from all participants prior to participation. Participants were compensated at a rate of JPY 1,000 per hour. One male actor and one female actor were recruited from the Himawari Theatre Group.

### Experimental setting

Participants completed an avatar-mediated dialogue task in a 2 × 2 between-subjects design crossing Actor Gender (female, male) and Real-Avatar type (cat, dog). Each participant interacted with either the male or the female actor while viewing two animated animal avatars side by side on a monitor (Figure 1). One avatar was animated in real time by the actor’s facial expressions and head movements (real avatar), whereas the other avatar displayed pre-recorded, non-contingent animation (fake avatar). For the fake avatar, one pre-recorded silent video was prepared for each avatar type. These videos showed natural facial and head movements intended to resemble those occurring during a real conversation, but they were not contingent on the ongoing dialogue. For each participant, either the cat or the dog avatar served as the real avatar, whereas the other avatar served as the fake avatar. Participants completed a single 8-min conversation. The dialogue followed a semi-structured outline on everyday topics. The same general topic sequence and approximate duration were used across participants, while limited improvisation was allowed to maintain natural conversation. Participants were informed only that the study examined eye movements during avatar-mediated communication and were not told about the non-contingent animation or the subsequent identification judgment. Participants were not informed of the actor’s gender before the conversation and obtained gender-related information from the actor’s voice during the dialogue. Immediately after the 8-min conversation, the experimenter entered the participant’s room and administered a post-experiment questionnaire. Participants were asked, “During the video call, the avatar actor’s movements were synchronized with one of the animal faces. Which animal do you think was synchronized with the actor?” They responded by selecting one of four options: cat, dog, both, or unsure. After responding to the question, participants were then fully debriefed and provided additional consent.

**Figure 1:**
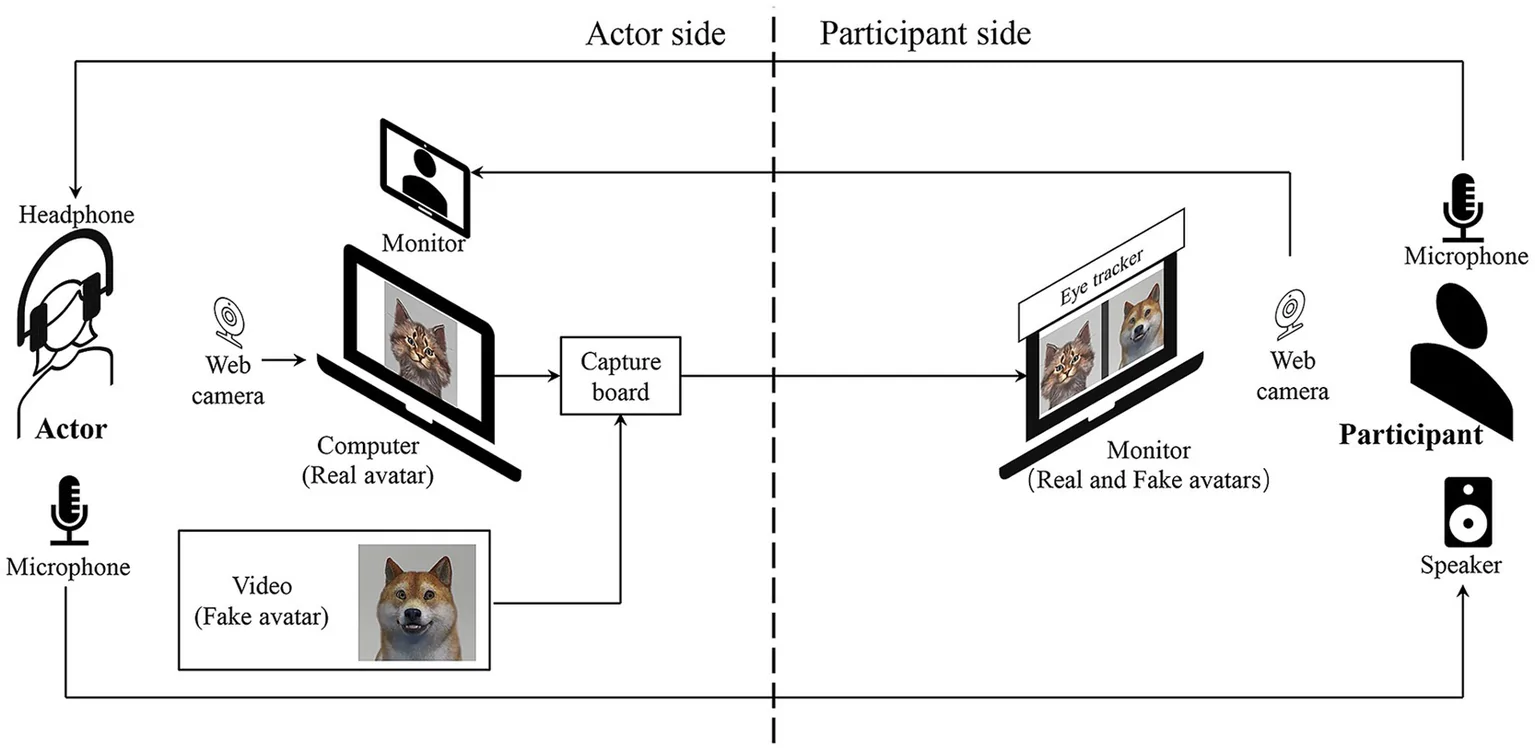
An example of schematic of the conversation-task setup in which the female actor controls the cat avatar (real avatar). The actor’s facial expressions and head movements are captured by a web camera and converted into the animated avatar on the actor-side computer, then streamed to the participant’s monitor. The pre-recorded video of the dog avatar (fake avatar) is also displayed on the participant’s monitor but moves non-contingently. The participant’s face is captured by a web camera and transmitted to the actor’s monitor. Audio communication is bidirectional via microphones and headphones/speakers. Participants’ eye movements are recorded using a Tobii Pro Spark eye tracker.

The participant and actor were seated in two adjacent, sound-isolated rooms connected via an online audiovisual system, with real-time bidirectional audio via microphones and headphones/speakers. On the actor side, facial movements were captured with a web camera and mapped onto the avatar in real time using FaceRig^©^ (Holotech Studios SRL, Romania). Before each conversation, the actor’s facial movements were calibrated to the avatar using the calibration function in FaceRig. The experimenter then visually confirmed that the actor’s mouth and head movements were appropriately synchronized with the avatar before starting the dialogue. The same calibration and confirmation procedure was applied across all actors and real-avatar conditions. The fake avatar was presented using pre-recorded video. The two avatar streams were composed and transmitted to the participant’s room using OBS Studio (OBS Project, https://obsproject.com/). Avatars were centered in the left and right halves of a 48 × 27 cm monitor (1,920 × 1,080) viewed at 60 cm (full-display visual angle: approximately 43.6° × 25.4°). Each avatar was shown within a circular region 9 cm in diameter (approximately 8.2°). To support natural interaction, the participant’s face was captured and streamed to the actor, whereas the participant viewed only the two avatars.

Across the four experimental groups, the left/right positions of the cat and dog avatars were counterbalanced across participants. Eye movements were recorded continuously with a Tobii Pro Spark eye tracker (Tobii Pro AB, Stockholm, Sweden; 60 Hz) mounted on the participant’s monitor and analyzed in Tobii Pro Lab (Tobii Pro AB, Version 1.217.49450). A five-point timed calibration with randomized target order was performed before recording, followed by calibration validation. Fixations were classified using the built-in Tobii I-VT (Fixation) filter. Noise reduction was performed using a three-sample moving median filter, with a 20-ms velocity-calculation window and a velocity threshold of 30°/s. Adjacent fixations were merged when separated by no more than 75 ms and 0.5°, and fixations shorter than 60 ms were discarded. Gap filling was disabled; therefore, missing gaze samples were not interpolated.

### Data analysis

Behavioral performance was classified as correct identification or misidentification. Selection of the real avatar was classified as correct identification, whereas selection of the fake avatar, both avatars, and “unsure” was classified as misidentification. Behavioral performance was then coded as correct identification (0) or misidentification (1) and analyzed using a binary logistic regression with Actor Gender (female, male), Real-Avatar Type (cat, dog), and their interaction as predictors. The Actor Gender × Real-Avatar Type interaction was evaluated using a likelihood-ratio test comparing the full model including the interaction with a reduced model containing only the two main effects. Odds ratios (ORs) with 95% confidence intervals (CIs) were reported as effect-size estimates.

For the eye-tracking analysis, fixation proportion was defined as fixation duration to a given avatar divided by the participant’s total valid fixation duration within the same segment. Thus, periods without valid fixation data did not contribute to the denominator. The proportion of successfully recorded gaze samples over the full recording was calculated for each participant as a measure of recording quality. The AOI for each avatar was defined as a 9-cm diameter circular region corresponding to the avatar’s face. Fixation proportions were computed separately for three consecutive time segments, each lasting approximately 160 s.

Prior to the mixed-design ANOVA, its assumptions were assessed. Residual normality was evaluated using Shapiro–Wilk tests and Q–Q plots. When the normality assumption was not satisfied, fixation proportions were logit-transformed. Zero fixation proportions were replaced with 0.001 before transformation to permit calculation of the logit. Normality was reassessed after transformation. Homogeneity of variance and sphericity were evaluated using Levene’s and Mauchly’s tests, respectively. The fixation proportion to the fake avatar was analyzed using a 3 × 2 × 2 mixed-design ANOVA with Time segment (Segment 1–3) as a within-subject factor and Actor gender (female, male) and Real-Avatar type (cat, dog) as between-subject factors. The ANOVA was conducted using IBM SPSS Statistics for Windows, Version 26.0 (IBM Corp., Armonk, NY, USA). Bonferroni adjustment was applied to pairwise comparisons of the Time segment main effect. Effect sizes for main and interaction effects were calculated as partial eta squared (ηp^2^) with 95% CIs. CIs were calculated in R (version 4.6.0) using a noncentral F-distribution approach implemented in the MBESS package (version 5.0.1; Kelley, 2007). The level of statistical significance was set at *p* < 0.05 in all analyses.

## Results

### Behavioral results

In the male actor × cat avatar group, one participant selected the fake avatar and one selected “both.” In the male actor × dog avatar group, two participants selected “both.” In the female actor × cat avatar group, one participant selected the fake avatar and one selected “both.” In the female actor × dog avatar group, six participants selected the fake avatar and two selected “both.” No participants selected “unsure.” All fake-avatar and “both” responses were classified as misidentification. With these responses classified as misidentifications, the numbers of correct and incorrect responses were 8/2, 8/2, 8/2, and 2/8. Overall behavioral performance across Actor Gender and Real-Avatar type is shown in Table 2. The behavioral performance was additionally visualized as the error rate using the same Actor Gender × Real-Avatar type structure (Figure 2A) to facilitate comparison with the eye-tracking analyses.

**Table 2:** Results of correct/incorrect judgments for experimental groups.

Real-Avatar typeActor gender		
	Cat	Dog
Male actor	Correct 8 Incorrect 2	Correct 8 Incorrect 2
Female actor	Correct 8 Incorrect 2	Correct 2 Incorrect 8

**Figure 2:**
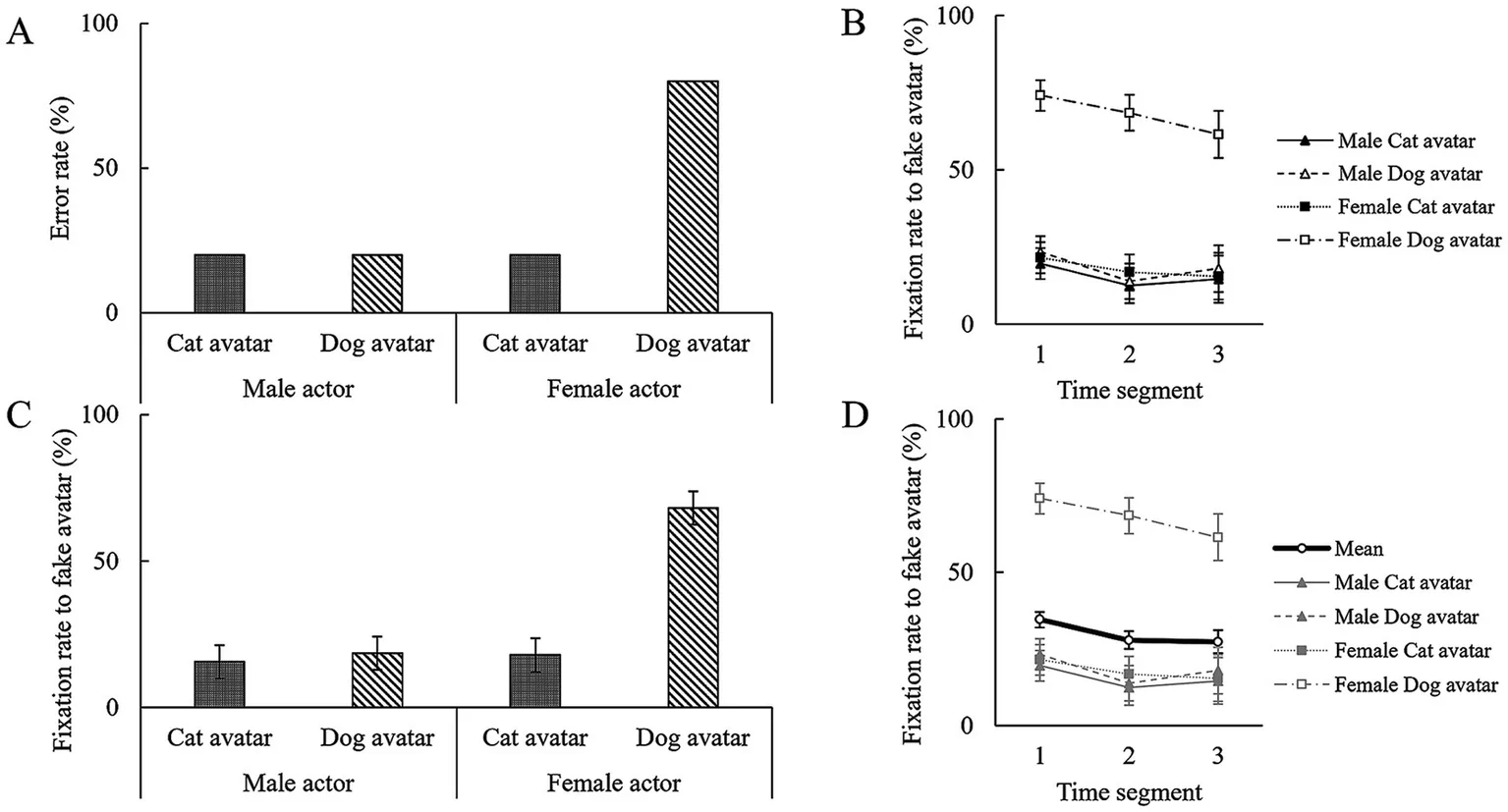
Error rates **(A)** and fixation rates to the fake avatar **(B–D)**. Rates of misidentification of the fake avatar as the real avatar **(A)** and fixation rates to the fake avatar of four groups against the time segments **(B)**. Error rates **(A)** and fixation rates to the fake avatar (**C**, see also Table 2) were prominently high in the group in which the dog avatar was controlled by a female actor. Fixation rates in response to the fake avatar decreased gradually in the later time segments **(D)**. Please note that the fixation data in this figure are untransformed; see the main text. Error bars: standard error.

The binary logistic regression showed that the Actor Gender × Real-Avatar Type interaction did not reach statistical significance (likelihood-ratio χ^2^ (1) = 3.167, *p* = 0.075, OR = 16.00, 95% CI [0.72, 354.80]). Descriptively, however, misidentification was most frequent in the female actor × dog real-avatar condition (8/10), whereas only 2/10 participants misidentified the fake avatar in each of the other three conditions.

### Eye-tracking results

Calibration was successfully completed for all participants. However, the validation data for one participant were no longer accessible; therefore, the following summary statistics are based on the remaining 39 participants. Mean validation accuracy was 0.56° (SD = 0.28°, range = 0.11–1.28°), and mean validation precision was 0.44° (SD = 0.30°, range = 0.11–1.67°), based on the SD measure provided by Tobii Pro Lab. Across participants, the proportion of successfully recorded gaze samples over the full recording was 80.95% (SD = 13.80%, range = 48–98%).

The residuals of the original fixation proportions deviated from normality; therefore, fixation proportions were logit-transformed. Following transformation, residual normality was satisfied in all three time segments (Shapiro–Wilk tests: all *p*s > 0.05), with no significant violations of homogeneity of variance (Levene’s tests, all *p* > 0.05) or sphericity (Mauchly’s *W* = 0.963, *p* > 0.05). For ease of interpretation, fixation proportions in Figure 2 are presented as untransformed percentages, whereas inferential statistics are based on logit-transformed data.

For fixation proportion to the fake avatar, the 3 × 2 × 2 mixed-design ANOVA revealed no significant Time segment × Real-Avatar type × Actor gender interaction (*F* [2, 72] = 0.373, *p* = 0.690, ηp^2^ = 0.010, 95% CI [0.000, 0.077]; Figure 2B).

A significant Actor gender × Real-Avatar type interaction was observed (*F* [1, 36] = 7.445, *p* = 0.010, ηp^2^ = 0.171, 95% CI [0.011, 0.387]). Simple-effect analyses showed that, within the female-actor conditions, fixation proportion to the fake avatar was higher in the dog-avatar condition than in the cat-avatar condition (*F* [1, 36] = 26.675, *p* < 0.001, ηp^2^ = 0.426, 95% CI [0.184, 0.605]). No corresponding effect of Real-Avatar type was observed within the male-actor conditions (*F* [1, 36] = 1.705, *p* = 0.200, ηp^2^ = 0.045, 95% CI [0.000, 0.230]; Figure 2C).

In addition, a significant main effect of Time segment was observed (F [2, 72] = 9.640, *p* < 0.001, ηp^2^ = 0.211, 95% CI [0.059, 0.361]; Figure 2D). Bonferroni-adjusted pairwise comparisons showed that fixation proportion to the fake avatar was higher in Segment 1 than in Segment 2 (*F* [1, 36] = 16.089, *p* < 0.001, ηp^2^ = 0.309, 95% CI [0.084, 0.513]) and Segment 3 (27.4%) (*F* [1, 36] = 15.168, *p* = 0.001, ηp^2^ = 0.296, 95% CI [0.076, 0.502]). No significant difference was observed between Segments 2 and 3 (*F* [1, 36] = 0.084, *p* = 1.000, ηp^2^ = 0.002, 95% CI [0.000, 0.112]).

## Discussion

The present study examined whether gender-related information conveyed by a communicator’s voice may be associated with animal-avatar appearance, with particular focus on the possible association between a female communicator and a cat avatar. Although the Actor Gender × Real-Avatar Type interaction did not reach statistical significance, misidentification was most frequent in the female actor × dog avatar condition (Table 2 and Figure 2A). In this condition, participants were more likely to identify the alternative cat avatar as the real communication partner and showed greater fixation to that fake avatar (Figure 2C). No comparable tendency was observed when the actor was male.

The higher misidentification rate in the female actor × dog avatar condition is consistent with the possibility that the cat-related neural response change reported by Murakami et al. (2024) reflected the pairing of a female communicator with a cat avatar rather than a general difference between cat and dog avatars. This pattern is consistent with the possibility that participants associated the female communicator more strongly with the cat avatar than with the dog avatar. Such an association is also compatible with previous research describing gendered impressions of cats and cat owners and cultural connections between cats and femininity (Dupuis and Girme, 2024; Lewis, 2024; Tu et al., 2024). The present findings therefore provide preliminary indirect evidence that gender-related vocal characteristics may influence the perception of animal-avatar appearance during avatar-mediated communication. This suggests that impressions of an avatar may depend partly on vocal information from the communicator, rather than on visual appearance alone.

The eye-tracking results complement the descriptive behavioral findings by showing that the higher misidentification rate in the female actor × dog avatar condition was also reflected in gaze allocation during the dialogue. Participants in this condition showed greater fixation to the fake cat avatar (Figure 2C). Fixation proportion to the fake avatar also decreased across the dialogue (Figure 2D). Descriptively, this decrease appeared more pronounced in the female actor × dog avatar condition than in the other conditions (Figure 2B). One possible interpretation is that participants initially expected the cat avatar to represent the female communicator but gradually attended less to it as its movements remained unrelated to the ongoing dialogue. Longer dialogue periods may be helpful for determining whether changes in gaze allocation are eventually accompanied by more accurate identification of the real communication partner.

Some limitations of the current study should be considered when interpreting the findings. First, actor gender and avatar type were each represented by single instances: one male actor, one female actor, one cat avatar, and one dog avatar. Thus, actor gender was confounded with the specific actors, and avatar type was confounded with the specific stimuli. The present design therefore cannot determine whether the observed results reflect general effects of communicator gender and avatar type or properties of the particular actors and avatars used in this study. Moreover, avatar appearance itself can convey gender-related impressions (Nowak and Rauh, 2005), and the cat and dog avatars used here were not independently evaluated for such impressions. Therefore, the present findings may indicate an association between the female communicator’s vocal characteristics and a cat avatar that happened to convey relatively feminine impressions, rather than a general association between female communicators and cat avatars per se. It is also possible that a dog avatar with similarly feminine characteristics could produce a comparable pattern. Accordingly, controlling the gender-related impressions of the avatars is important for determining whether the observed association reflects animal type itself or the specific gendered appearance of the avatar. Second, participants’ preferences for cats and dogs were not assessed. Such preferences may have influenced both gaze allocation and identification of the real communication partner independently of expectations about the communicator. Finally, the relatively small sample size limited the statistical power of behavioral analysis. Although a pronounced descriptive difference was observed, with correct identification in 8 of 10 participants in each of the other three groups but only 2 of 10 participants in the female actor × dog avatar group, the Actor Gender × Real-Avatar Type interaction did not reach statistical significance. Nevertheless, the present eye-tracking analysis detected an interaction between avatar appearance and gender-related vocal characteristics that was not clearly captured by the behavioral responses. Future studies using a larger sample, together with multiple communicators and multiple cat and dog avatars, should assess participants’ preferences for cats and dogs to determine whether the present findings can be observed across different communicators and avatar appearances.

The present findings suggest that replacing a communicator’s real appearance with an animal avatar does not necessarily eliminate expectations about the communicator. Rather, gender-related vocal characteristics may interact with avatar appearance and influence how the communicator is perceived. This issue may be particularly relevant in multi-user avatar-mediated settings, including VR-based group therapy, where participants interact through personalized avatars and voices (Skiers et al., 2025). In such settings, a mismatch between vocal characteristics and avatar appearance could potentially increase ambiguity in identifying or distinguishing communication partners. The present study therefore highlights a previously underexamined issue arising from the combination of voice and avatar appearance in increasingly diverse avatar-mediated social environments. The findings suggest that associations between communicator gender and animal-avatar appearance may influence how communication partners are identified during avatar-mediated interaction.

## Data Availability

The raw data supporting the conclusions of this article will be made available by the authors, without undue reservation.

## References

[ref1] Banakou D. Kishore S. Slater M. (2018). Virtually being Einstein results in an improvement in cognitive task performance and a decrease in age Bias. Front. Psychol. 9:917. doi: 10.3389/fpsyg.2018.00917, 29942270 PMC6004376

[ref2] Dupuis H. E. Girme Y. U. (2024). “Cat ladies” and “mama’s boys”: a mixed-methods analysis of the gendered discrimination and stereotypes of single women and single men. Personal. Soc. Psychol. Bull. 50, 314–328. doi: 10.1177/01461672231203123, 37876182 PMC10860362

[ref3] Faul F. Erdfelder E. Buchner A. Lang A.-G. (2009). Statistical power analyses using G*power 3.1: tests for correlation and regression analyses. Behav. Res. Methods 41, 1149–1160. doi: 10.3758/BRM.41.4.1149, 19897823

[ref4] Fong K. Mar R. A. (2015). What does my avatar say about me? Inferring personality from avatars. Personal. Soc. Psychol. Bull. 41, 237–249. doi: 10.1177/0146167214562761, 25576173

[ref5] Guerra E. Bernotat J. Carvacho H. Bohner G. (2021). Ladies first: gender stereotypes drive anticipatory eye-movements during incremental sentence interpretation. Front. Psychol. 12:589429. doi: 10.3389/fpsyg.2021.589429, 34276460 PMC8279744

[ref6] Hansen B. C. Rakhshan P. J. Ho A. K. Pannasch S. (2015). Looking at others through implicitly or explicitly prejudiced eyes. Vis. Cogn. 23, 612–642. doi: 10.1080/13506285.2015.1063554

[ref7] Hoogerbrugge A. J. Hooge I. T. C. Hessels R. S. Strauch C. (2025). When is enough enough? Empirical guidelines to determine participant sample size for scene viewing studies. Behav. Res. 57:241. doi: 10.3758/s13428-025-02754-8, 40721900 PMC12304073

[ref8] Kelley K. (2007). Confidence intervals for standardized effect sizes: theory, application, and implementation. J. Stat. Softw. 20:1–24. doi: 10.18637/jss.v020.i08

[ref9] Kobayashi Y. Kawakami T. Matsumoto S. Yoshihisa T. Teranishi Y. Shimojo S. (2021) How Do avatar appearances affect communication from others? 2021 IEEE 45th Annual Computers, Software, and Applications Conference (COMPSAC), 1036–1039

[ref10] Lau K. H. C. Stark P. Bozkir E. Kasneci E. (2026) Skin-Deep Bias: How Avatar Appearances Shape Perceptions of AI Hiring Proceedings of the 2026 CHI Conference on Human Factors in Computing Systems (CHI ‘26), 1–20

[ref11] Lewis R. A. (2024). Feline entanglements: feminist interspecies care and solidarity in a post-pandemic world. Hypatia 39, 795–811. doi: 10.1017/hyp.2024.13

[ref12] Machneva M. Evans A. M. Stavrova O. (2022). Consensus and (lack of) accuracy in perceptions of avatar trustworthiness. Comput. Hum. Behav. 126:107017. doi: 10.1016/j.chb.2021.107017

[ref13] Mack K. Hsu R. C. L. Monroy-Hernández A. Smith B. A. Liu F. (2023) Towards inclusive avatars: disability representation in avatar platforms. Proceedings of the 2023 CHI Conference on Human Factors in Computing Systems Hamburg ACM 1–13.

[ref14] Menshikova G. Y. Saveleva O. A. Zinchenko Y. P. (2018). The study of ethnic attitudes during interactions with avatars in virtual environments. Psych. Rus. 11, 20–31. doi: 10.11621/pir.2018.0102

[ref15] Murakami Y. Han Y. Watanabe H. Shimojo A. Yokosawa K. (2024). Brain activity in face-processing regions evoked by avatars and avatar-mediated communication. Bull. Jpn. Health Care Univ. 10, 11–22. doi: 10.11422/jscn.54.7

[ref16] Nowak K. L. Rauh C. (2005). The influence of the avatar on online perceptions of anthropomorphism, androgyny, credibility, homophily, and attraction. J Comp Mediated Comm 11, 153–178. doi: 10.1111/j.1083-6101.2006.tb00308.x

[ref17] Peck T. C. Seinfeld S. Aglioti S. M. Slater M. (2013). Putting yourself in the skin of a black avatar reduces implicit racial bias. Conscious. Cogn. 22, 779–787. doi: 10.1016/j.concog.2013.04.016, 23727712

[ref18] Saragih J. M. Lucey S. Cohn J. F. (2011) Real-time avatar animation from a single image 2011 IEEE International Conference on Automatic Face & Gesture Recognition (FG) IEEE 213–220 10.1109/FG.2011.5771383 PMC3935737 24598812

[ref19] Skiers K. Peng D. Kundu A. Person T. Takase K. Nagoshi T. . (2025). “I was truly able to express the image of myself that I have within”: exploring VR Group therapy approaches with the LGBTQIA+ community. IEEE Trans. Vis. Comput. Graph. 31, 9688–9698. doi: 10.1109/TVCG.2025.3616754, 41052124

[ref20] Tu A. Y. Springer C. M. Albright J. D. (2024). Evaluation of characteristics associated with self-identified cat or dog preference in pet owners and correlation of preference with pet interactions and care: an exploratory study. Animals 14:2534. doi: 10.3390/ani14172534, 39272319 PMC11394057

[ref21] Zhang S. Faruk O. Porzel R. Küster D. Schultz T. Liu H. (2024) Examining the effects of human-likeness of avatars on emotion perception and emotion elicitation 2024 International Conference on Activity and Behavior Computing (ABC) Oita IEEE 1–12

